# Expression of Myomaker and Myomerger in myofibers causes muscle pathology

**DOI:** 10.1186/s13395-023-00317-z

**Published:** 2023-05-01

**Authors:** Phillip C. Witcher, Chengyi Sun, Douglas P. Millay

**Affiliations:** 1 Division of Molecular Cardiovascular Biology, Cincinnati Children’s Hospital Medical Center, Cincinnati, OH USA; 2 Department of Pediatrics, University of Cincinnati College of Medicine, Cincinnati, OH 45229 USA

**Keywords:** Myomaker, Myomerger/Myomixer, Muscle pathology, Myocyte fusion

## Abstract

**Background:**

Skeletal muscle development and regeneration depend on cellular fusion of myogenic progenitors to generate multinucleated myofibers. These progenitors utilize two muscle-specific fusogens, Myomaker and Myomerger, which function by remodeling cell membranes to fuse to each other or to existing myofibers. Myomaker and Myomerger expression is restricted to differentiating progenitor cells as they are not detected in adult myofibers. However, Myomaker remains expressed in myofibers from mice with muscular dystrophy. Ablation of Myomaker from dystrophic myofibers results in reduced membrane damage, leading to a model where persistent fusogen expression in myofibers, in contrast to myoblasts, is harmful.

**Methods:**

Dox-inducible transgenic mice were developed to ectopically express Myomaker or Myomerger in the myofiber compartment of skeletal muscle. We quantified indices of myofiber membrane damage, such as serum creatine kinase and IgM^+^ myofibers, and assessed general muscle histology, including central nucleation, myofiber size, and fibrosis.

**Results:**

Myomaker or Myomerger expression in myofibers independently caused membrane damage at acute time points. This damage led to muscle pathology, manifesting with centrally nucleated myofibers and muscle atrophy. Dual expression of both Myomaker and Myomerger in myofibers exacerbated several aspects of muscle pathology compared to expression of either fusogen by itself.

**Conclusions:**

These data reveal that while myofibers can tolerate some level of Myomaker and Myomerger, expression of a single fusogen above a threshold or co-expression of both fusogens is damaging to myofibers. These results explain the paradigm that their expression in myofibers can have deleterious consequences in muscle pathologies and highlight the need for their highly restricted expression during myogenesis and fusion.

**Supplementary Information:**

The online version contains supplementary material available at 10.1186/s13395-023-00317-z.

## Introduction

Skeletal muscle is comprised of multinucleated myofibers formed from the fusion of activated satellite cells, the resident stem cell of skeletal muscle. During development, satellite cells differentiate into myocytes and then fuse to each other to form the skeletal muscle syncytium [1–3]. Myomaker and Myomerger are two muscle-specific fusogens necessary for this fusion process during development and regeneration [4–7]. Myomaker and Myomerger are membrane active proteins that function independently at distinct points of the fusion pathway [8]. While Myomaker functions at or before the hemifusion step of the pathway, where lipids of the outer leaflet of the plasma membrane mix, Myomerger drives pore formation and fusion completion. Myomaker has seven transmembrane domains and an indispensable palmitoylated C-terminal cytoplasmic tail [9]. Although Myomaker shares structural characteristics with lipid hydrolases, its precise activity that confers hemifusion competence is not understood [10–13]. Myomerger, in contrast, is a single-pass transmembrane protein with two extracellular *α*-helical domains that insert in membranes causing destabilizations needed for formation of fusion pores [14, 15].

While absolutely essential for muscle regeneration, expression of these muscle fusogens is highly regulated and specific to the myoblast stage [16]. Their expression is not detected in myofibers after fusion, and genetic data indicates that transcription of the *Myomaker* gene is dispensable in myofibers for their fusion with progenitor cells [17, 18]. Moreover, Myomaker expression in myofibers during muscle overload and dystrophic disease is contributed from fusion of progenitor cells indicating that myonuclei within the myofiber lack the ability to transcribe Myomaker [18, 19], further highlighting the degree to which the expression of these fusogens is transcriptionally restricted. Stringent control of Myomaker and Myomerger expression is likely needed due to their inherent membrane-remodeling activities. Indeed, Myomerger helices within its ectodomain insert in membranes to convert hemifusion events to full fusion [14, 15]. Given Myomaker is needed to achieve cell hemifusion, a thermodynamically unfavorable event [20], it is plausible that it, too, may have activity which remodels the plasma membrane. We propose that the consequences of these membrane-remodeling effects by the fusogens could be deleterious in certain cell types such as myofibers where they are not normally expressed. This concept is supported by evidence in which Myomaker was genetically deleted in dystrophic myofibers resulting in a reduction of membrane damage [18]. Through ectopic expression of the fusogens in otherwise normal myofibers, we sought to further test the model that consequences of their membrane-remodeling effects could be independently deleterious.

In this study, we assessed the impact of Myomaker and Myomerger activity within the myofiber compartment. To study the fusogens in an in vivo setting, transgenic mice were generated to ectopically express Myomaker or Myomerger within myofibers using a doxycycline-inducible system. We found that both fusogens can individually impact myofiber membrane integrity. When expressed together, muscle pathology was exacerbated compared to expression of either fusogen by itself. Altogether, these data support a paradigm whereby Myomaker and Myomerger, while necessary for myoblast fusion, can independently contribute to muscle pathology when expressed in mature skeletal myofibers, even in the absence of dystrophin-deficiency.

## Results

### Development of an inducible model for ectopic fusogen expression in myofibers

**Fig. 1:**
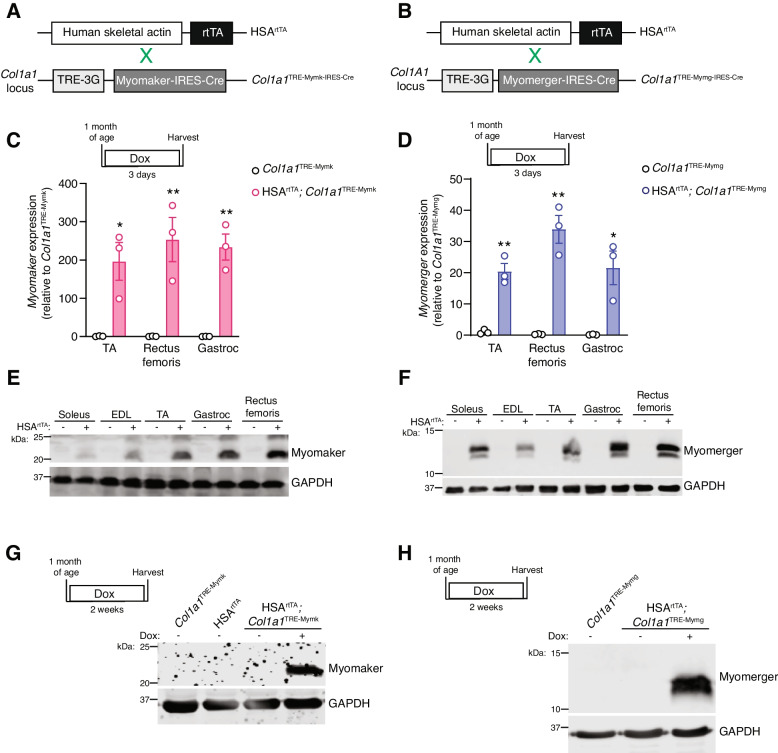
Validation of transgenic models to activate Myomaker and Myomerger expression in myofibers. **A** Schematic of the breeding strategy for inducible expression of Myomaker within myofibers. **B** Schematic of the breeding strategy for inducible expression of Myomerger within myofibers. **C** qPCR analysis of *Myomaker* mRNA levels from the TA, rectus femoris, and gastroc muscles after 3 days of dox treatment. **D** qPCR analysis for *Myomerger* mRNA levels from the TA, rectus femoris, and gastroc muscles after 3 days of induction. **E** Western blot for Myomaker expression in the soleus, EDL, TA, gastroc, and rectus femoris after 3 days of dox treatment. **F** Western blot for Myomerger expression in the soleus, EDL, TA, gastroc, and rectus femoris after 3 days of dox treatment. **G** Western blot for Myomaker expression in the gastroc muscle after 2 weeks of induction. **H** Western blot for Myomerger in the gastroc after 2 weeks of induction. Statistical analyses and presentation: data are presented as mean ± SEM. **C** and **D** Two-tailed Student’s *t*-test; *P < 0.05, **P < 0.01

To ectopically induce expression of the muscle fusogens in the myofiber compartment, we employed a doxycycline-inducible system. Each gene was independently inserted downstream of a tetracycline response element (TRE) at the *Col1a1* locus (Fig. 1A, B). This construct also contained Cre recombinase downstream of Myomaker or Myomerger, linked by an internal ribosome entry site (IRES). These mice were crossed with a previously generated transgenic mouse which utilizes the human skeletal *α*-actin (HSA) promoter to drive expression of reverse tetracycline transactivator (rtTA) [21]. The resulting mouse lines, HSA^rtTA^; *Col1a1*^TRE−Mymk−IRES−Cre^ (iMymk) and HSA^rtTA^; *Col1a1*^TRE−Mymg−IRES−Cre^ (iMymg), allowed for both temporal and spatial control of Myomaker or Myomerger expression in myofiber compartments of skeletal muscle. To assess induction of the fusogens in various muscles, *Myomaker* and *Myomerger* mRNA was measured in the tibialis anterior (TA), rectus femoris, and gastrocnemius (gastroc) muscles after a 3-day induction with doxycycline chow (Fig. 1C, D). Protein expression of Myomaker and Myomerger was validated in the soleus, extensor digitorum longus (EDL), TA, gastroc, and rectus femoris muscles after 3 days of induction (Fig. 1E, F). Controls used for 1C-F were dox-treated *Col1a1*^TRE−Mymk^ or *Col1a1*^TRE−Mymg^ mice, which lacked HSA^rtTA^. We also confirmed that the mouse models did not have leaky expression of Myomaker or Myomerger in the absence of doxycycline in the gastroc (Fig. 1G,H).

### Myomaker expression in myofibers leads to membrane damage and muscle pathology

**Fig. 2:**
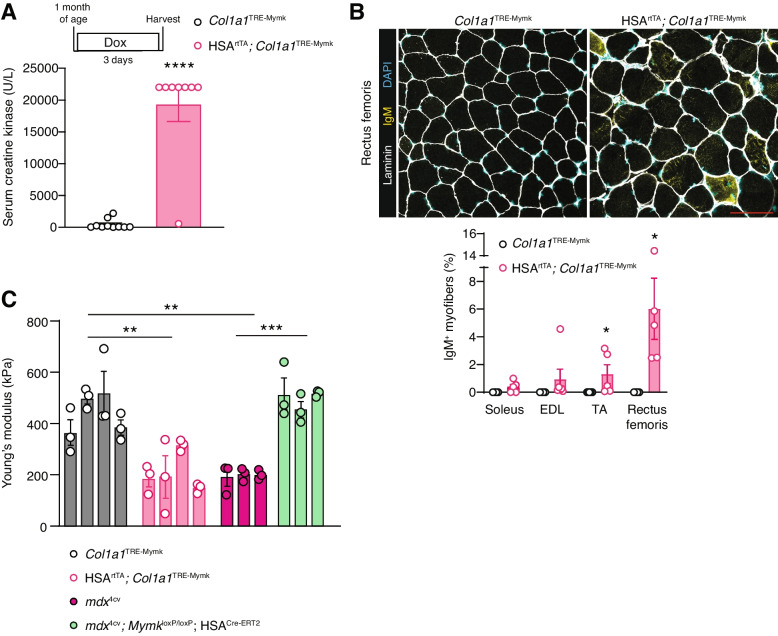
Myomaker expression in myofibers is associated with myofiber membrane damage and reduced myofiber stiffness. **A** Serum CK levels are elevated after 3 days of Myomaker induction in myofibers. **B** Representative images of immunofluorescence staining for IgM in the rectus femoris after 3 days of Myomaker induction reveal an elevated proportion of IgM^+^ myofibers compared to the control. Quantification of the percentage of IgM^+^ myofibers in the soleus, EDL, TA, and rectus femoris is shown below the images. Scale bar = 100 μm. **C** Atomic force microscopy on isolated myofibers reveals reduced myofiber stiffness with Myomaker expression in myofibers 3 days after induction. Myofiber stiffness in dystrophic myofibers is restored to wild-type levels after ablation of Myomaker from myofibers. Each bar represents the average stiffness of three myofibers from a given mouse. Statistical analyses and presentation: data are presented as mean ± SEM. **A** and **B** Two-tailed Student’s *t*-test; *P < 0.05, ****P < 0.0001. **C** One-way ANOVA between experimental groups with a Tukey’s post hoc test; **P < 0.01, ***P < 0.001

Gene expression downstream of the TRE element has been validated as soon as 24 h after induction with doxycycline with the HSA promoter [21]. Thus, we first wanted to assess the impact of short-term Myomaker expression in the myofiber compartment. Evidence of myofiber membrane damage was present as early as 3 days after induction, as shown by the elevated serum creatine kinase (CK) (Fig. 2A) and increased proportion of IgM^+^ myofibers (Fig. 2B). The proportion of IgM^+^ myofibers in each muscle appeared to correlate with the level of Myomaker expression (Figs. 1E and 2B). Previous studies report that dystrophic myofibers exhibit an altered response to an atomic force microscopy (AFM)-based indentation probe, which is generally interpretated as a reduction in myofiber stiffness [22]. Gene-mediated rescue of dystrophic myofibers restores myofiber stiffness, suggesting that a cause of disrupted myofiber architecture in dystrophic myofibers is the lack of dystrophin [23]. To characterize if Myomaker in myofibers impacts the biophysical properties of myofibers, such as stiffness, we performed AFM on control and iMymk myofibers. We observed that after 3 days of induction, Myomaker expression within myofibers caused a reduction in myofiber stiffness (Fig. 2C). These data are consistent with the concept that Myomaker may disrupt the myofiber membrane and alter its biophysical properties.

Consistent with previous reports, we also observed reduced stiffness of dystrophic myofibers (Fig. 2C), which was unexpectedly comparable to that of myofibers with ectopic Myomaker expression. Since we previously showed that a reduction of Myomaker in dystrophic myofibers leads to more stable myofiber membranes, we also wanted to evaluate stiffness in this model. We deleted Myomaker in myofibers by treating *mdx*^4cv^; *Mymk*^loxP/loxP^; HSA^CreERT2 18^ with tamoxifen starting at 2 months of age. Ablating Myomaker from dystrophic myofibers resulted in a normalization of myofiber stiffness (Fig. 2C). Overall, these data reveal the deleterious effects of Myomaker in both wild-type and dystrophic myofibers.

**Fig. 3:**
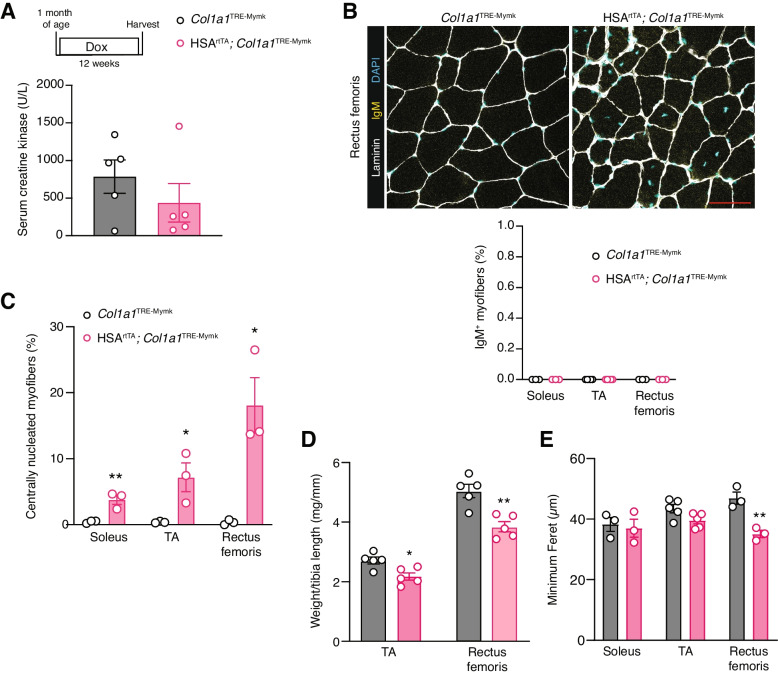
Long-term Myomaker expression in myofibers leads to elevated centrally nucleated myofibers and muscle atrophy. **A** Serum CK levels are not elevated after 12 weeks of Myomaker expression in myofibers. **B** Immunofluorescence staining for IgM in the rectus femoris after 12 weeks of Myomaker induction. Quantification of the percentage of IgM^+^ myofibers in the soleus, TA, and rectus femoris is shown below the images. Scale bar = 100 μm. **C** Quantification of myofibers with centrally localized nuclei in the soleus, TA, and rectus femoris reveals elevated levels of regeneration with Myomaker expression in myofibers. **D** Muscle mass to tibia length ratios of the rectus femoris and TA were reduced after 12 weeks of Myomaker expression in myofibers. **E** Myofiber size, quantified by minimum Feret’s diameter, was quantified in the soleus, TA, and rectus femoris. Statistical analyses and presentation: data are presented as mean ± SEM. **C**-**E** two-tailed Student’s *t*-test; *P < 0.05, **P < 0.01

We next wanted to evaluate the long-term effects of Myomaker expression in myofibers. We sacrificed mice 12 weeks after activation of Myomaker in myofibers, and, surprisingly, we did not observe direct signs of myofiber membrane damage based on levels of creatine kinase in the serum or IgM^+^ myofibers (Fig. 3A, B). The lack of detectable membrane damage could be due to reduced levels of Myomaker expression after 12 weeks of induction compared to 3 days (Fig. S1 A, B). Despite this apparent reduction in Mymk levels, we detected central nuclei (Fig. 3C), a marker of myofiber repair, reduced muscle masses (Fig. 3D), and reduced myofiber size in the rectus femoris (Fig. 3E) after 12 weeks of Myomaker expression. Altogether, these data implicate Myomaker as a contributor to muscle pathology. We interpret the pathological effects in the long-term to be a result of Myomaker-induced membrane damage at an early stage after activation of the transgene.

### Myomerger expression is damaging to skeletal myofibers causing altered muscle histology

Because myoblast fusion also requires Myomerger and this protein could be activated in muscle pathologies, given its similar expression pattern to Myomaker in normal muscle [4, 5], we wanted to test the effects of Myomerger in the myofiber compartment. No evidence of membrane damage or changes in muscle mass were observed following a 3-day induction of Myomerger in myofibers (Fig. S2A–C). Even after 8 weeks of Myomerger induction within myofibers, myofiber membrane damage was absent, and no changes in muscle mass or histology were observed (Fig. S2 D–F).

These data conflicted with an alternative approach to assessing the impact of Myomerger within myofibers. Given its high transduction efficiency in skeletal muscle, we utilized adeno-associated virus serotype 9 (AAV9) with a CMV promoter to drive expression of Myomerger in skeletal muscle [24]. AAV9-Myomerger or AAV9-GFP (control) was intramuscularly injected in the TA of a 2-month-old wild-type mice, and muscle was harvested 2 weeks after the injection. Successful transduction of AAV9-Myomerger was confirmed by Western blot analysis (Fig. S3A). Despite the level of Myomerger protein, at the level of the whole muscle, being lower compared to the iMymg model (Fig. S3B), central nucleation was observed 2 weeks after injection of AAV9-Myomerger, suggesting damage and subsequent regeneration (Fig. S3C). To resolve why regeneration was observed with viral transduction of skeletal muscle but not the myofiber-inducible model, despite lower levels of Myomerger in the AAV system, we performed immunofluorescence for Myomerger to determine expression at the level of individual myofibers. We found that expression on a per myofiber basis was significantly higher with AAV9-Myomerger compared to the inducible model (Fig. S3D). We interpret the Myomerger-negative myofibers in the AAV system to be derived from fusion of progenitors that were not transduced with AAV9. These data associate Myomerger with induction of regeneration and suggest that myofibers may have a threshold of Myomerger expression which they can tolerate.

**Fig. 4:**
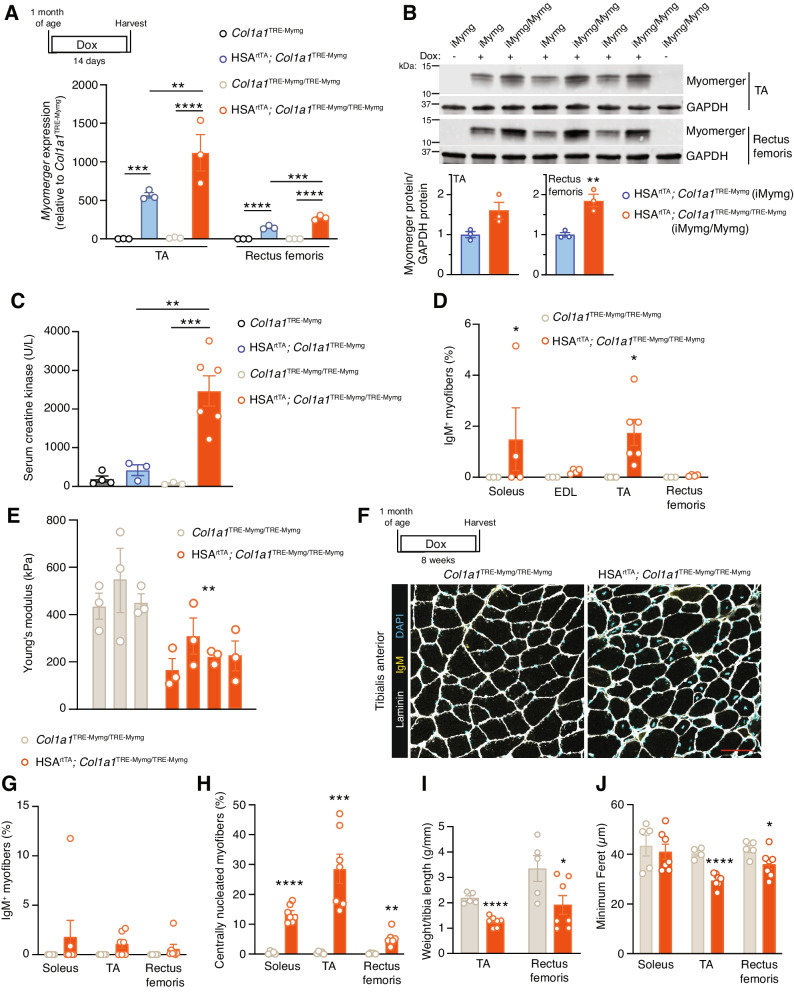
Muscle pathology results from elevated Myomerger expression in myofibers. **A** qPCR analysis reveals elevated levels of *Myomerger* in the TA and rectus femoris muscles of homozygous mice compared to heterozygous mice after 2 weeks of induction. **B** Quantification of Western blots demonstrates elevated Myomerger protein content in the TA and rectus femoris muscles of iMymg/Mymg mice compared to iMymg mice after 2 weeks of induction. **C** Serum CK levels in iMymg/Mymg mice are elevated after 2 weeks of Myomerger expression. Levels are also significantly higher than 2 weeks of Myomerger expression in iMymg mice. **D** Quantification of the proportion of IgM^+^ myofibers reveals elevated levels of damage in the soleus and TA muscles after 2 weeks of Myomerger expression. **E** Myofiber stiffness is reduced after 2 weeks of Myomerger expression in myofibers by atomic force microscopy. Each bar represents the average stiffness of three myofibers from a given mouse. **F** Representative sections of the TA muscle from iMymg/Mymg mice after 8 weeks of Myomerger expression revealed a lack of significantly elevated proportion of IgM^+^ myofibers (**G**). **H** Elevated levels of centrally nucleated myofibers were observed in the soleus, TA, and rectus femoris. Scale bar = 100 μm. **I** Muscle mass to tibia length ratios were reduced in the TA and rectus femoris after 8 weeks of Myomerger expression in myofibers. **J** Myofiber size, quantified by minimum Feret’s diameter, was quantified in the soleus, TA, and rectus femoris. Statistical analyses and presentation: data are presented as mean ± SEM; **A** and **C** One-way ANOVA with a Tukey’s post hoc test compared samples from the same muscle; *P < 0.05, **P < 0.01, ***P < 0.001, ****P < 0.0001. **B**, **D**–**E**, and **G**–**J** two-tailed Student’s *t* test, identical muscles were compared in **D** and **G**–**J**; stiffness values were compared between the two groups using average values from each mouse for **E**; *P < 0.05, **P < 0.01, ***P < 0.001, ****P < 0.0001

We genetically increased expression of Myomerger in the myofiber with the inducible transgenic mice. Mice homozygous for the *Col1a1*^TRE−Mymg−IRES−Cre^ transgene (iMymg/Mymg) had higher *Myomerger* mRNA levels compared to hemizygous mice (Fig. 4A). Myomerger protein was also elevated in the TA and rectus femoris muscles of iMymg/Mymg mice compared to iMymg mice (Fig. 4B). Several indices of myofiber membrane damage were elevated after 14 days of induction, the earliest timepoint where changes in serum CK (Fig. 4C) and the proportion of IgM^+^ myofibers (Fig. 4D) were detected, suggesting that the higher myofiber expression of Myomerger can indeed result in myofiber membrane damage. Using the previously described AFM-based indentation approach, we found that the stiffness of these myofibers was reduced compared to the control, indicating that damage from Myomerger levels and activity may impact myofiber stiffness (Fig. 4E). Assessing if Myomerger had a long-term effect in myofibers, we observed altered pathology (Fig. 4F), but the proportion of IgM^+^ myofibers was not significantly changed compared to the control (Fig. 4G). We also observed an increase in regeneration, reduced muscle mass indices, and reduced myofiber size (Fig. 4H–J). Overall, these data reveal that accumulation of Myomerger above certain threshold levels negatively impacts myofiber membrane integrity.

### Dual Myomaker and Myomerger expression in myofibers exacerbates muscle pathology

**Fig. 5:**
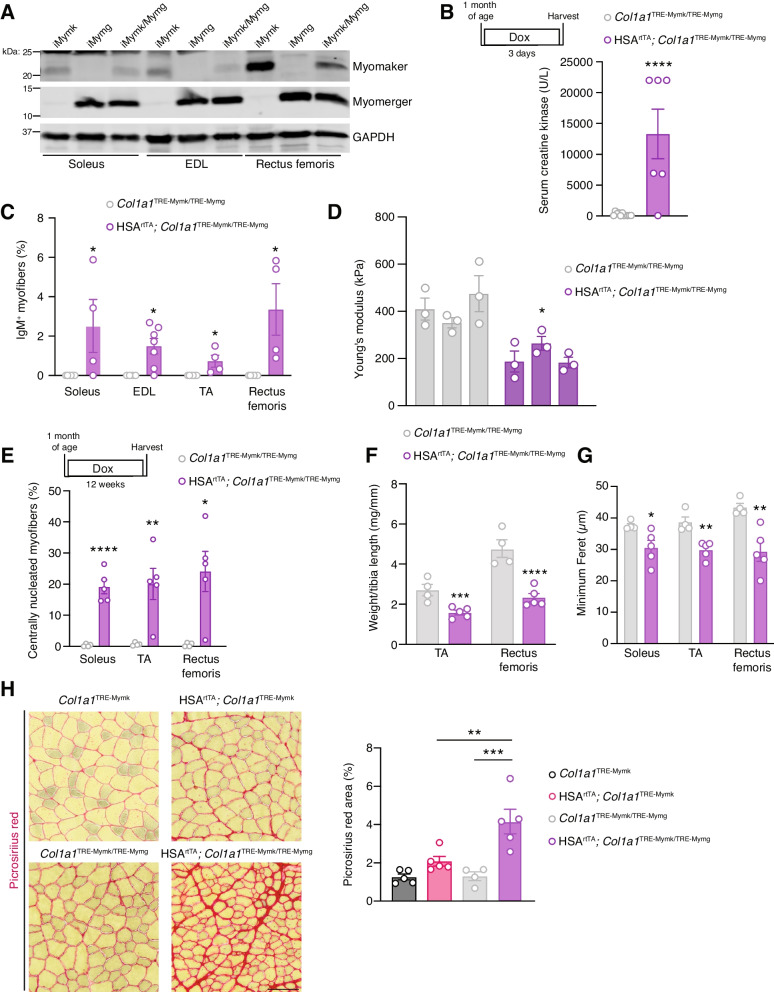
Expression of both Myomaker and Myomerger in myofibers results in combinatorial effects. **A** Western blots for Myomaker and Myomerger expression in the soleus, EDL, and rectus femoris after 3 days of dox treatment. **B** Serum CK levels after 3 days of induction in myofibers. **C** Quantification of the proportion of IgM^+^ myofibers in the soleus, EDL, TA, and rectus femoris after 3 days of induction reveals an elevated proportion of IgM^+^ myofibers compared to the control in all four muscles. **D** Myofiber stiffness assessed by atomic force microscopy is reduced after 3 days of concurrent Myomaker and Myomerger expression. Each bar represents the average stiffness of three myofibers from a given mouse. **E** Quantification of myofibers with centrally localized nuclei in the soleus, TA, and rectus femoris. **F** Muscle mass to tibia length ratios of the TA and rectus femoris. **G** Myofiber size in the soleus, TA, and rectus femoris, quantified by minimum Feret’s diameter. **H** Picrosirius red staining revealed elevated fibrosis when both Myomaker and Myomerger are expressed in myofibers. Scale bar = 100 μm. Fibrosis is quantified as the percentage of total area staining positive for Picrosirius red. Statistical analyses and presentation: Data are presented as mean ± SEM; **B**–**G** Two-tailed Student’s *t*-test, identical muscles were compared in **C** and **E–G**; stiffness values were compared between the two groups using average values from each mouse for **D**; *P < 0.05, **P < 0.01, ***P < 0.001, ****P < 0.0001. **H** one-way ANOVA with a Tukey’s post hoc test; **P < 0.01, ***P < 0.001

Thus far, we have demonstrated that Myomaker and Myomerger are individually capable of negatively impacting the myofiber membrane and leading to muscle pathology. However, they are normally expressed together as myoblasts are fusing to repair damaged myofibers or generating new myofibers [17, 25]. To study the impact of Myomaker and Myomerger co-expression in myofibers, we crossed the two inducible mouse lines to generate HSA^rtTA^; *Col1a1*^TRE−Mymk−IRES−Cre^, *Col1a1*^TRE−Mymg−IRES−Cre^ (iMymk/Mymg). *Myomaker* mRNA levels in the iMymk/Mymg mouse were reduced from that of the iMymk mouse (Fig. S4A), but the level of reduction was not statistically significant. Myomaker protein content also appeared slightly lower (Fig. 5A). Reduced mRNA levels were observed for *Myomerger* in iMymk/iMymg muscle compared to the iMymg mouse (Fig. S4A), but such differences were not observed at the protein level (Fig. 5A). Since we have already established that this level of Myomerger does not elicit effects on muscle pathology (Fig. S2), this model allows us to test the combinatorial effects of Myomaker and Myomerger activities. The iMymk/Mymg mouse exhibited increased levels of membrane damage compared to control mice after 3 days of dox treatment (Fig. 5B,C). When assessing the stiffness of these fibers, we found that it was reduced compared to the control (Fig. 5D), similar to reductions observed in iMymk (Fig. 2C) and iMymg/Mymg (Fig. 4E) myofibers.

Downstream pathology was evident in iMymk/iMymg mice after 12 weeks of dox treatment (Fig. S4B). Due to the lack of myofiber membrane damage or downstream consequences on muscle pathology in the iMymg mouse (Fig. S2), indices of pathology in the iMymk/Mymg mice were only compared to iMymk mice. For centrally nucleated myofibers, in the iMymk mice, we observed 7.2% in the TA, 18.1% in the rectus femoris, and 3.8% in the soleus (Fig. 3C), but the iMymk/Mymg mice exhibited 20% in the TA, 24% in the rectus femoris, and 19.2% in the soleus (Fig. 5E). Effects on muscle masses and myofiber sizes were also exacerbated in iMymk/iMymg mice. iMymk mice exhibited muscle mass reductions of 20% in the TA and 24% in the rectus femoris (Fig. 3D), whereas iMymk/iMymg mice displayed reductions of 42% in the TA and 52% in the rectus femoris (Fig. 5F). The average myofiber size was reduced by 3% in the soleus, 9% in the TA, and 25% in the rectus femoris of iMymk mice (Fig. 3E) and by 18% in the soleus, 23% in the TA, and 32% in the rectus femoris of iMymk/iMymg mice (Fig. 5G). Consistent with the concept of an exacerbated phenotype when both fusogens are expressed in myofibers, we detected increased fibrosis in iMymk/iMymg muscle (Fig. 5H). These data indicate that ectopic co-expression of Myomaker and Myomerger in myofibers has a combinatorial impact on muscle pathology.

## Discussion

In this study, we sought to evaluate the consequences of Myomaker and Myomerger within the terminally differentiated units of skeletal muscle, myofibers. We show that short-term induction of Myomaker in the myofiber compartment led to compromised myofiber integrity, which aligns with previous work that linked the myofiber activity of Myomaker to dystrophic pathology [18]. Acute expression of Myomerger similarly led to compromised myofiber integrity. This loss of integrity triggered increased pathology in both inducible models, including centrally nucleated fibers and reduced muscle mass indices. In sum, we postulate that expression of the fusogens in the short-term yields membrane damage causing detectable changes in muscle histology in the long-term. These data are consistent with the concept that expression of the muscle fusogens needs to be highly restricted during myoblast fusion to prevent negative outcomes within the myofiber.

Despite the unambiguous damaging consequences of Myomaker and Myomerger in the myofiber compartment, careful analysis of the data reveals that wild-type myofibers have a threshold for which they may tolerate fusogen expression. Myofiber membrane damage was not observed after induction of Myomerger until expression was genetically increased (Fig. 4). The same paradigm holds true for Myomaker, where lower levels of Myomaker after 12 weeks of expression compared to 3 days are not sufficient to induce detectable myofiber membrane damage (Fig. 3B). While this study implicates the fusogens as having a negative effect on myofibers, it does not definitively discount the possibility that Myomaker and Myomerger could have functional roles on the myofiber for fusion in some contexts.

When Myomaker and Myomerger were co-expressed, indices of myofiber membrane damage, muscle regeneration, and muscle atrophy were exacerbated. When Myomerger was expressed alone, no detectable levels of serum CK or IgM^+^ myofibers were observed (Fig. S2). However, when the same level of Myomerger was expressed with Myomaker, elevated levels of IgM^+^ myofibers were observed in the short term, and increased centrally nucleated myofibers, muscle atrophy, and fibrosis were observed in the long term (Fig. 5). These data are consistent with the paradigm that these two fusogens have independent but overlapping membrane remodeling activities, which drive fusion in myoblasts but cause membrane instability in myofibers.

To evaluate the biophysical consequences of Myomaker and Myomerger in the myofiber, we utilized an AFM-based indentation approach to measure myofiber stiffness. Myofiber stiffness is primarily a function of two parameters: cytoskeletal components and plasma membrane integrity. Actin and myosin are the main cytoskeletal contributors to skeletal muscle stiffness, while cholesterol and lipid saturation are membrane contributors [26, 27]. Cell stiffness is associated with changes in function, increasing as myocytes differentiate to myotubes [26]. Although previous studies utilizing AFM-based indentation to quantify skeletal muscle stiffness have reported variable stiffness values for skeletal muscle, absolute stiffness values measured by AFM indentation protocols are strongly dependent on the experimental parameters, such as the model and the method used to analyze the results [28–31]. For example, cell fixation with PFA increases the measured stiffness [32]. Despite divergent quantifications of cell stiffness, there is a general consensus that dystrophic muscle is less stiff than its wild-type counterpart [22, 23, 33]. The reason behind this difference has previously been attributed to the loss of structural integrity provided by dystrophin, a crucial protein linking intracellular cytoskeletal components to the basal lamina [34, 35]. Here, we provide evidence that reduced myofiber stiffness in dystrophy may not be primarily caused by lack of dystrophin. The activity of the muscle fusogens, essential for myoblast fusion during regeneration, individually led to reduced myofiber stiffness, and ablation of Myomaker in dystrophic myofibers restored stiffness to a level comparable to that of wild-type myofibers. These data support the concept that ectopic fusogen expression and activity in myofibers may contribute to reduced stiffness in dystrophic myofibers.

Defective function of myogenic progenitors has been implicated in muscular dystrophy pathology. Although repetitive rounds of degeneration and regeneration lead to an exhausted satellite cell pool [36–38], other studies have shown that an increased number of satellite cells are present in dystrophic muscle [39, 40]. Despite this discordance, it is apparent that dystrophic satellite cells exhibit impaired regenerative potential [41, 42]. The inability to replace necrotic myofibers culminates in fibro-fatty replacement of skeletal muscle and muscle atrophy [43, 44]. Collectively, those data indicate that the regenerative process goes awry in dystrophy, which could overall accelerate pathology. However, there is increasing evidence that the regenerative program has maladaptive features during skeletal muscle disease [45, 46]. Indeed, ablation of satellite cells in dystrophic mouse models results in a situation where remaining myofibers exhibit increased size and stabilized membranes [47]. Based on this work, one could envision a scenario where reduction of satellite cell activity could be a valuable therapeutic approach. However, in the long-term, ablation of satellite cells or blockade of their fusogenic activity results in muscle wasting in a dystrophic setting [18]. Interestingly, attenuation of the MyoD pathway in dystrophic myofibers blunts sarcolemma instability [47], which is consistent with a maladaptive function in this setting for Myomaker and Myomerger given that these proteins are transcriptionally induced by MyoD. Thus, instead of broadly modulating satellite cells and their corresponding regenerative capacity that has beneficial consequences for long-term muscle maintenance, specifically targeting negative consequences of chronic fusion, namely persistent delivery of progenitor-derived Myomaker and Myomerger to myofibers, could be an approach to mitigate pathology in dystrophic tissue.

One limitation of our study is that we are only able to detect robust membrane damage through IgM analysis and serum CK levels. These methods of assessing myofiber membrane damage may not detect more moderate levels of damage. This is exemplified by elevated levels of centrally nucleated myofibers despite initially low proportions of IgM^+^ myofibers in the iMymk soleus (Figs. 2B and 3C) and iMymg/Mymg rectus femoris (Fig. 4D, H). Thus, the analytical pipelines used to determine loss of membrane integrity were not able to stratify the deleterious effects of the fusogens. Additionally, the nature of membrane damage caused by Myomaker and Myomerger may not be identical. Another limitation of the our study is that we are unable to uncouple the potential activity of the fusogens at the plasma membrane and intracellular compartments. Previous studies have demonstrated that Myomaker not only resides at the plasma membrane but also in the Golgi and post-Golgi vesicles [48]. Additionally, Myomerger has been shown to also associate with intracellular membrane compartments [5]. Overexpression of either fusogen could accordingly have a negative impact in other membrane-bound organelles, which perhaps could explain why we observed a strong atrophy phenotype. Our study was also limited by the lack of measurements for the same muscle. For example, myofiber stiffness was not quantified for the TA or rectus femoris due to inherent challenges with isolating individual myofibers from these muscles, and we did not assess long-term pathology in the EDL, which was used for AFM measurements. However, there is a consistent pattern of elevated levels of myofiber membrane damage early (measured by the proportion of IgM^+^ myofibers and serum CK) and muscle pathology in the long-term (elevated centrally nucleated myofibers and muscle atrophy).

In summary, this study supports a paradigm whereby Myomaker and Myomerger are essential for fusion of muscle progenitors but have deleterious consequences within myofibers. The adverse effects of Myomaker and Myomerger in myofibers could explain why their expression is so tightly restricted to the myocyte stage of the muscle lineage. Persistent and dysregulated activation of the regeneration program in skeletal muscle may lead to unintended consequences of these membrane-active fusogens disrupting myofiber membranes, further exacerbating myofiber membrane damage in pathologic conditions, like muscular dystrophy. Downregulation of these fusogens in myofibers may serve as a potential therapeutic option for reducing muscle damage in muscular dystrophy.

## Methods

### Mice

This study was performed entirely in mice using either commercially available transgenic mice or novel transgenic mice generated as described below. All mice used in this study were maintained on a *C57BL/6* background. For ectopic expression of the muscle fusogens, doxycycline-inducible transgenes, TRE3G-Myomaker-IRES2-Cre-pA, and TRE3G-Myomerger-IRES2-Cre-pA were targeted into the Col1a1 safe harbor (CaSH) locus using a CRISPR/Cas9-mediated approach developed by Transgenic Animal and Genome Editing Core at Cincinnati Children’s Hospital Medical Center. The transgenes were inserted to a genetic location ~ 1.65 kb downstream of the Col1a1 gene in a reverse orientation. This was achieved using a sgRNA (target sequence: GGGAGGAAACCTGCCCTTGG) and a donor plasmid containing the transgene flanked with the 5′ and 3′ homologous arms at the length of 2.5 kb and 3.0 kb, respectively. The donor plasmids were amplified and purified with the EndoFree Plasmid Kit (Qiagen). The targeted transgenic mice were generated via pronuclear injection of fertilized C57BL/6 eggs with Cas9 protein (IDT, Catalog no. 1081059), synthetic sgRNA (Synthego), and the donor plasmid at a concentration of 40 ng/µl, 20 ng/µl, and 4 ng/µl, respectively. The injected eggs were transferred immediately into the oviductal ampulla of pseudopregnant CD-1 females for development and birth. The pups were then genotyped by long-range PCR and Sanger sequencing. These mice (*Col1a1*^TRE−Mymk IRES−Cre^ and *Col1a1*^TRE−Mymg IRES−Cre^) were crossed with mice carrying the HSA^rtTA^ allele to drive fusogen expression in the myofiber compartment [21]. Dual expression of Myomaker and Myomerger was generated by breeding the *Col1a1*^TRE−Mymk IRES−Cre^ mice with *Col1a1*^TRE−Mymg IRES−Cre^ mice, followed by breeding with the HSA^rtTA^ mouse. Myofiber-specific deletion of *Mymk* in the dystrophic background was accomplished by introducing an HSA^CreERT2^ allele into the *Mymk*^loxP/loxP^ *mdx*^4cv^ mouse [18, 49].

To induce fusogen expression in myofibers, 1- to 2-month-old mice were provided chow supplemented with 0.0625% doxycycline (TestDiet). Tissue was collected immediately upon completion of doxycycline treatment.

Tamoxifen (MilliporeSigma) was prepared in corn oil with 10% ethanol at a concentration of 25 mg/mL. Mice were given intraperitoneal injections of tamoxifen (0.075 mg/kg/d) for 4 days to induce recombination. For experiments with the HSA^CreERT2^ allele, mice were then maintained on tamoxifen by injection every third day.

AAV9-GFP and AAV9-Myomerger were generated by Vigene Biosciences and intramuscularly injected (5 × 10^11^ genome copies/injection, diluted with sterile PBS) into the TA muscle of 2-month-old mice while under inhaled isoflurane anesthesia. The injection site was prepared by first removing hair with hair clippers and then sanitizing the area with chlorhexidine gluconate and allowing it to dry.

### Muscle collection and sample preparation

Mouse hindlimb muscles were dissected, dried, and weighed. Tibias were dissected, and remaining tissue was digested with proteinase K (0.4 mg/mL) overnight at 55 °C, after which tibia length was measured using digital calipers. Muscles were embedded in 10% tragacanth/PBS (MilliporeSigma) and frozen in 2-methylbutane cooled in liquid nitrogen. We used 10-μm sections for all histology. For RNA and immunoblot preparations, tissues were flash frozen in liquid nitrogen immediately upon collection.

### Histological analyses

Immunohistochemical studies were performed as described previously with minor modifications [18]. Briefly, sections were fixed in 1% PFA/PBS and permeabilized with 0.2% Triton X-100/PBS. Sections were blocked using 2% BSA, 1% heat-inactivated goat serum, and 0.1% Tween-20/PBS. Primary antibodies were incubated overnight at 4 °C, and secondary Alexa Fluor antibodies (1:300) were applied at room temperature for 30 min. Anti-laminin antibody (1:300, MilliporeSigma, stock no. L9393) was used to visualize the outline of all myofibers present in each muscle section. IgM primary antibody conjugated to Texas Red (1:100, MilliporeSigma, stock no. SAB3701210) was used to highlight myofibers with compromised membrane integrity. Anti-ESGP antibody (1:100, R&D, stock no. AF4580) was used to stain Myomerger protein on muscle sections. Immunostained slides were imaged using a Nikon A1R confocal system. Centrally located myonuclei were quantified from two 10 × images using ImageJ (NIH). IgM-positive myofibers and myofiber size were quantified from the entire muscle section using NIS-Elements software (Nikon).

Picrosirius red staining was used to quantify muscle fibrosis. Briefly, fresh-frozen sections were incubated overnight in Bouin’s solution. After a 5-min wash in PBS, sections were incubated in working Weigert’s hematoxylin for 5 min before a 1-h incubation in picrosirius red. Sections were dipped two times in 0.5% acetic acid and three times in ethanol. Three 1-min exchanges in xylenes were performed before mounting. Picrosirius red-stained sections were imaged using an Olympus BX60 widefield microscope. Fibrosis was quantified from two 10 × images using ImageJ (NIH).

Gross pathology was assessed with hematoxylin and eosin (H&E) staining. Fresh-frozen sections were incubated in 10% formalin for 5 min before washing in PBS for 2 min followed by a 2-min wash in tap water. After incubating sections in working Weigert’s hematoxylin for 5 min, they were rinsed with tap water until tap water ran clear. Sections were dipped ten times in 0.7% eosin Y, ten times in 95% ethanol, ten times in 95% ethanol, ten times in 100% ethanol, ten times in 100% ethanol, ten times in xylene, ten times in xylene, and ten times in one last xylene solution before mounting. H&E stained sections were imaged using an Olympus BX60 widefield microscope. All image analyses were performed in a blinded fashion.

### RNA analysis

Total RNA was isolated from muscle samples using established TRIzol protocols (Life Technologies, stock no. 15596018). cDNA was synthesized with the Superscript VILO cDNA Synthesis Kit (Invitrogen, Thermo Fishers Scientific, stock no. 11754250). Standard qPCR methods were used with PowerUp SYBR Green Master Mix (Applied Biosystems, Thermo Fisher Scientific), and the assay was performed on the Bio-Rad CFX96 Real-Time System with the following primers: *GAPDH*: forward, 5′-TGCGACTTCAACAGCAACTC-3′; reverse, 5′-GCCTCTCTTGCTCAGTGTCC-3′, *Mymk*: forward, 5′-ATCGCTACCAAGAGGCGTT-3′; reverse, 5′-CACAGCACAGACAAACCAGG-3′, and *Mymx*: forward, 5′-CAGGAGGGCAAGAAGTTCAG-3′; reverse, 5′-ATGTCTTGGGAGCTCAGTCG-3′. mRNA levels were quantified using the *ΔΔ*Ct method [50].

### Western blotting

After measuring the mass, muscles were homogenized in muscle lysis buffer (10-mM Tris, 1-mM EDTA, 0.5% Triton X-100, and 50-mM NaF buffer, pH 7.4) supplemented with a protease inhibitor cocktail (Sigma Aldrich, stock no. 5056489001). Solubilization was allowed to proceed on a nutator for 2 h at 4 °C. Protein lysates were prepared for SDS-PAGE analysis by heating at 95 °C for 5 min in 1 × Laemmli sample buffer containing 10% beta-mercaptoethanol. Proteins were resolved on discontinuous polyacrylamide gels (12% for Myomaker and 15% for Myomerger) and transferred to Immobilon-FL PVDF membranes (Millipore Sigma, stock no. IPFL00010). Membranes were blocked in 5% milk/TBST for 1 h at room temperature before incubation with primary antibodies in 5% BSA/TBST against Myomaker (1:250, provided from Dr. Leonid Chernomordik laboratory), Myomerger (1:200, R&D, stock no. AF4580), and GAPDH (1:5000, Millipore, stock no. MAB374) overnight on a nutator at 4 °C. The resulting immunoblots generated after incubation with relevant secondary antibodies (goat anti-rabbit IgG DyLight 800, Cell Signaling Technology, stock no. 5151; donkey anti-Sheep IgG Alexa Fluor 680, Invitrogen Thermo Fisher Scientific, stock no. A21102; goat anti-mouse IgG DyLight 680, Cell Signaling Technology, stock no. 5470; goat anti-mouse IgG Dylight 800, Cell Signaling Technology, stock no. 5257) were scanned, imaged, and analyzed using the Odyssey CLx imaging system (LI-COR Biosciences, stock no. 9140). Protein expression was quantified using densitometric analysis tools on ImageJ (NIH). The band intensities of Myomaker and Myomerger were measured and standardized to the intensity of the housekeeping gene, GAPDH.

### Serum creatine kinase

Serum creatinine kinase levels were measured using a Roche c 311 clinical chemistry analyzer per manufacturer instructions.

### Atomic force microscopy

Atomic force microscopy was used to measure stiffness of single muscle fibers. For isolation of single muscle fibers, whole EDL muscles were incubated in 0.22% type 1 collagenase (MilliporeSigma C0130) in DMEM at 37 °C for 40 min. Following incubation, muscles were triturated in PBS to release individual myofibers. The myofibers were subsequently washed with PBS before fixing in 4% PFA/PBS for 10 min at room temperature, after which the fixed fibers were washed again with PBS and stored at 4 °C. Fixed myofibers were placed on double-sided tape applied to the bottom of 60-mm plates. The plates were centrifuged at 400 g for 10 min at room temperature to attach the myofibers to the tape [29]. Attached myofibers were submerged in 0.22-µm-filtered PBS prior to measurement by AFM. Stiffness was quantified using the contact mode of force mapping on a NanoWizard 4 XP BioScience atomic force microscope with a HybridStage (Bruker). A Nikon Eclipse Ti-U inverted microscope permitted precise positioning of the cantilever tip above the myofiber. Before each experiment, the cantilever was calibrated while submerged in PBS in a region of the dish that did not contain a myofiber nearby. A z-closed loop with constant force, 0.05-nN set point, 1.0-μm z length, 2.0 μm/s z speed, and 0.0-s contact time, was used to make sixty-four measurements were collected from a 10 × 10 µm area of the myofiber. The calibrated spring constant of cantilever D was used to convert the photodiode signal into a force value (*k*_nom_ = 0.03 N/m, MLCT-BIO; Bruker). Young’s modulus was extracted from each force-indentation curve using a modified Hertz model with the Bilodeau formula for a quadratic pyramidal indenter [51]:

$$F=0.7453\frac{E}{1-{v}^{2}}{\delta }^{2}\mathrm{tan}\,\alpha,$$

where *F* is the indentation force, *E* is Young’s modulus, *v* is Poisson’s ratio (approximated as 0.5, the value for isotropic incompressible materials), *δ* is the indentation (vertical tip position), and *α* is the half face angle of the pyramid (17.5° for cantilever D). The equivalent radius of a contact circle was calculated as the following:

$${a}_{e}=0.709\delta \mathrm{tan}\,\alpha,$$

where *a*_e_ is the equivalent radius of contact circle, *δ* is the indentation (vertical tip position), and *α* is the half face angle of the pyramid (17.5° for cantilever D). The data curve was fitted using a least squares fit with the Levenberg–Marquardt algorithm. The contact point, baseline, and Young’s modulus values were all fitted simultaneously. Measurements were taken at three different locations and averaged to yield the stiffness of a given myofiber. The mean stiffness of three unique myofibers comprised the myofiber stiffness for a given mouse.

### Statistics

All statistical analysis was performed using GraphPad Prism 9 software. Data are presented as mean ± standard error of the mean. Groups were assessed for normality using a Shapiro–Wilk test and analyzed using a one-way ANOVA with post hoc Tukey’s for multiple comparisons. Significant differences between two groups were determined using a two-tailed unpaired Student’s *t*-test. Statistical significance throughout was set at *P*-values less than 0.05. Specific statistical tests are noted in the figure legends.

## Supplementary Information

**Additional file 1: Figure S1.** Myomaker expression is lower after twelve weeks of induction compared to three days of induction. A qPCR analysis of *Myomaker* mRNA levels from the TA and rectus femoris muscles after twelve weeks or three days of induction. B Quantification of Western blot for Myomaker expression in the TA and rectus femoris relative to GAPDH expression demonstrates reduced Myomaker protein after twelve weeks of induction compared to three days of induction. Statistical analyses and presentation: Data are presented as mean ± SEM; A one-way ANOVA with a Tukey’s post hoc test compared samples from the same muscle; ***P < 0.001, ****P < 0.0001; B two-tailed Student’s t test; ** P < 0.01. **Figure S2.** Myomerger expression in myofibers of iMymg mice does not lead to pathology. A Serum CK levels are not elevated after three days of Myomerger expression in myofibers. B Immunofluorescence staining for IgM after three days of Myomerger induction. Quantification of the percentage of IgM^+^ myofibers in the soleus, TA, and rectus femoris is shown below the images. Scale bar = 100 μm. C Muscle mass to tibia length ratios of the TA and rectus femoris are not altered after three days of Myomerger expression in myofibers. D Serum CK levels are not elevated after eight weeks of Myomerger expression in myofibers. E Immunofluorescence staining for IgM after eight weeks of Myomerger induction. Quantification of the percentage of IgM^+^ myofibers in the soleus, TA, and rectus femoris is shown below the images. Scale bar = 100 μm. F Muscle mass to tibia length ratios of the TA and rectus femoris muscles are not altered after eight weeks of Myomerger expression in myofibers. Statistical analyses and presentation: Data are presented as mean ± SEM; A,C,D,F two-tailed Student’s t test (within the same muscle for C and F). **Figure S3.** Elevated Myomerger expression leads to muscle regeneration. A Western blot for Myomerger validated transduction by AAV9-Myomerger two weeks after IM injection in the TA. B Western blot of Myomerger revealed higher levels of Myomerger in iMymg muscle after two weeks of dox treatment compared to AAV9-Myomerger injected muscle two weeks after IM injection. C Histological analysis revealed elevated levels of centrally nucleated myofibers in the TA two weeks after IM injection with AAV9-Myomerger. Scale bar = 100 μm. D Myomerger immunofluorescence staining of the TA revealed elevated levels of Myomerger after AAV9-Myomerger (two weeks post-injection) compared to the myofiber inducible mouse model after two weeks of dox treatment. Fluorescence intensity of Myomerger antibody staining is quantified in arbitrary units (AU). Scale bar = 100 μm. Statistical analyses and presentation: Data are presented as mean ± SEM; C two-tailed Student’s t test; *P < 0.05; D one-way ANOVA with a Tukey’s post hoc test; ***P < 0.001, ****P < 0.0001. **Figure S4.** Myomaker and Myomerger expression in myofibers leads to muscle pathology. A qPCR analysis comparing *Myomaker* and *Myomerger* mRNA levels in the gastroc muscle between iMymk, iMymg, and iMymk/Mymg mice after three days of induction. B Representative H&E sections from the TA of *Col1a1*^TRE-Mymk/TRE-Mymg^ and HSA^rtTA^; *Col1a1*^TRE-Mymk/TRE-Mymg^ mice. Scale bar = 200 μm. Statistical analyses and presentation: Data are presented as mean ± SEM; A one-way ANOVA with a Tukey’s post hoc test; **P < 0.01, ****P < 0.0001.

## Data Availability

Materials reported here are available upon request.
