# Supplementary material for: Expression of Myomaker and Myomerger in myofibers causes muscle pathology
Source: Skelet Muscle. 2023 May 1;13:8. doi: 10.1186/s13395-023-00317-z (PMC10150476; doi:10.1186/s13395-023-00317-z)
Supplement: Supplementary file 1 — Additional file 1: Figure S1. Myomaker expression is lower after twelve weeks of induction compared to three days of induction. A qPCR analysis of Myomaker mRNA levels from the TA and rectus femoris muscles after twelve weeks or three days of induction. B Quantification of Western blot for Myomaker expression in the TA and rectus femoris relative to GAPDH expression demonstrates reduced Myomaker protein after twelve weeks of induction compared to three days of induction. Statistical analyses and presentation: Data are presented as mean ± SEM; A one-way ANOVA with a Tukey’s post hoc test compared samples from the same muscle; ***P < 0.001, ****P < 0.0001; B two-tailed Student’s t test; ** P < 0.01. Figure S2. Myomerger expression in myofibers of iMymg mice does not lead to pathology. A Serum CK levels are not elevated after three days of Myomerger expression in myofibers. B Immunofluorescence staining for IgM after three days of Myomerger induction. Quantification of the percentage of IgM+ myofibers in the soleus, TA, and rectus femoris is shown below the images. Scale bar = 100 μm. C Muscle mass to tibia length ratios of the TA and rectus femoris are not altered after three days of Myomerger expression in myofibers. D Serum CK levels are not elevated after eight weeks of Myomerger expression in myofibers. E Immunofluorescence staining for IgM after eight weeks of Myomerger induction. Quantification of the percentage of IgM+ myofibers in the soleus, TA, and rectus femoris is shown below the images. Scale bar = 100 μm. F Muscle mass to tibia length ratios of the TA and rectus femoris muscles are not altered after eight weeks of Myomerger expression in myofibers. Statistical analyses and presentation: Data are presented as mean ± SEM; A,C,D,F two-tailed Student’s t test (within the same muscle for C and F). Figure S3. Elevated Myomerger expression leads to muscle regeneration. A Western blot for Myomerger validated transduction by AAV9-Myomerger two weeks after IM inje [file 13395_2023_317_MOESM1_ESM.docx]

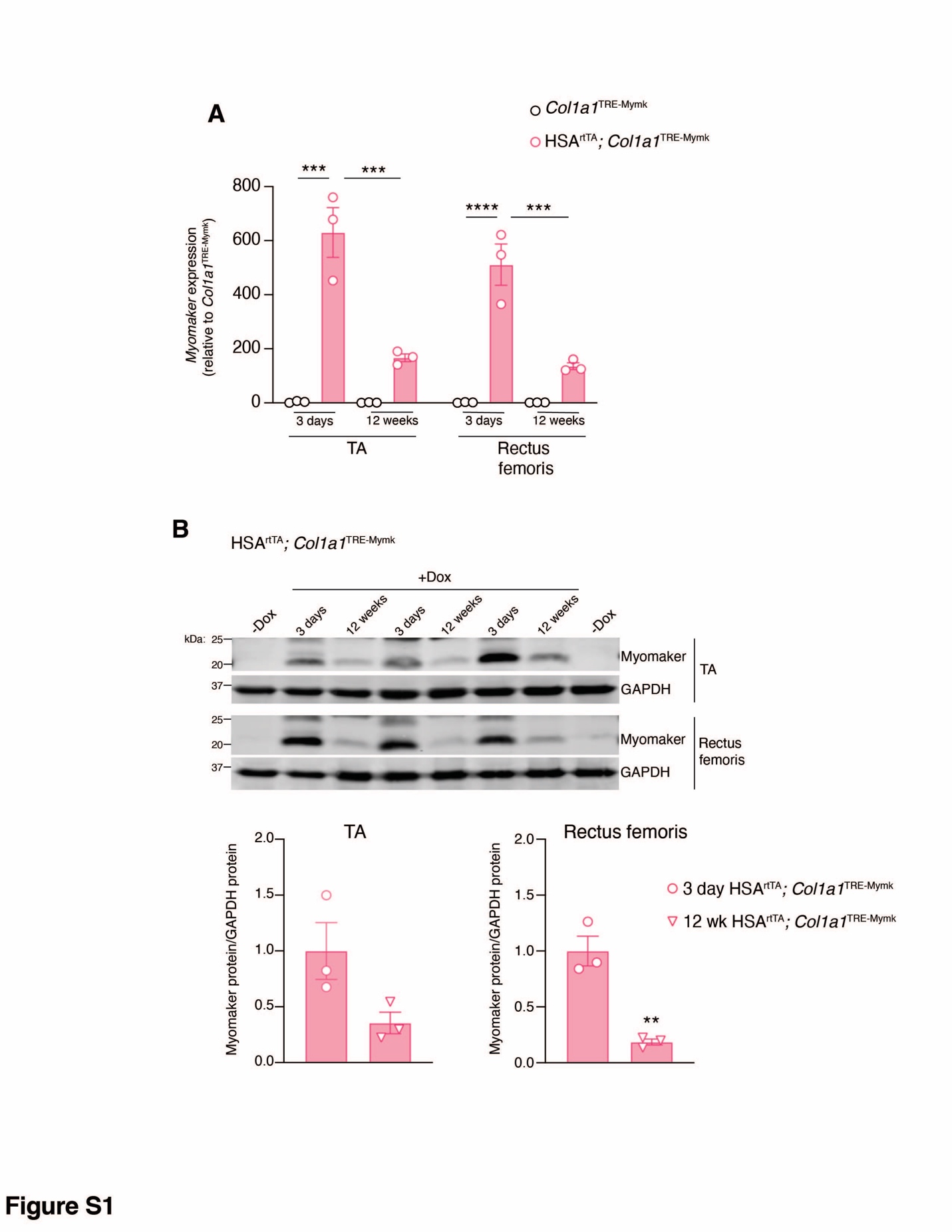


**Figure S1.** Myomaker expression is lower after twelve weeks of induction compared to three days of induction. **A** qPCR analysis of *Myomaker* mRNA levels from the TA and rectus femoris muscles after twelve weeks or three days of induction. **B** Quantification of Western blot for Myomaker expression in the TA and rectus femoris relative to GAPDH expression demonstrates reduced Myomaker protein after twelve weeks of induction compared to three days of induction. Statistical analyses and presentation: Data are presented as mean ± SEM; **A** one-way ANOVA with a Tukey’s post hoc test compared samples from the same muscle; ****P* < 0.001, *****P* < 0.0001; **B** two-tailed Student’s *t* test; ** *P* < 0.01.

**
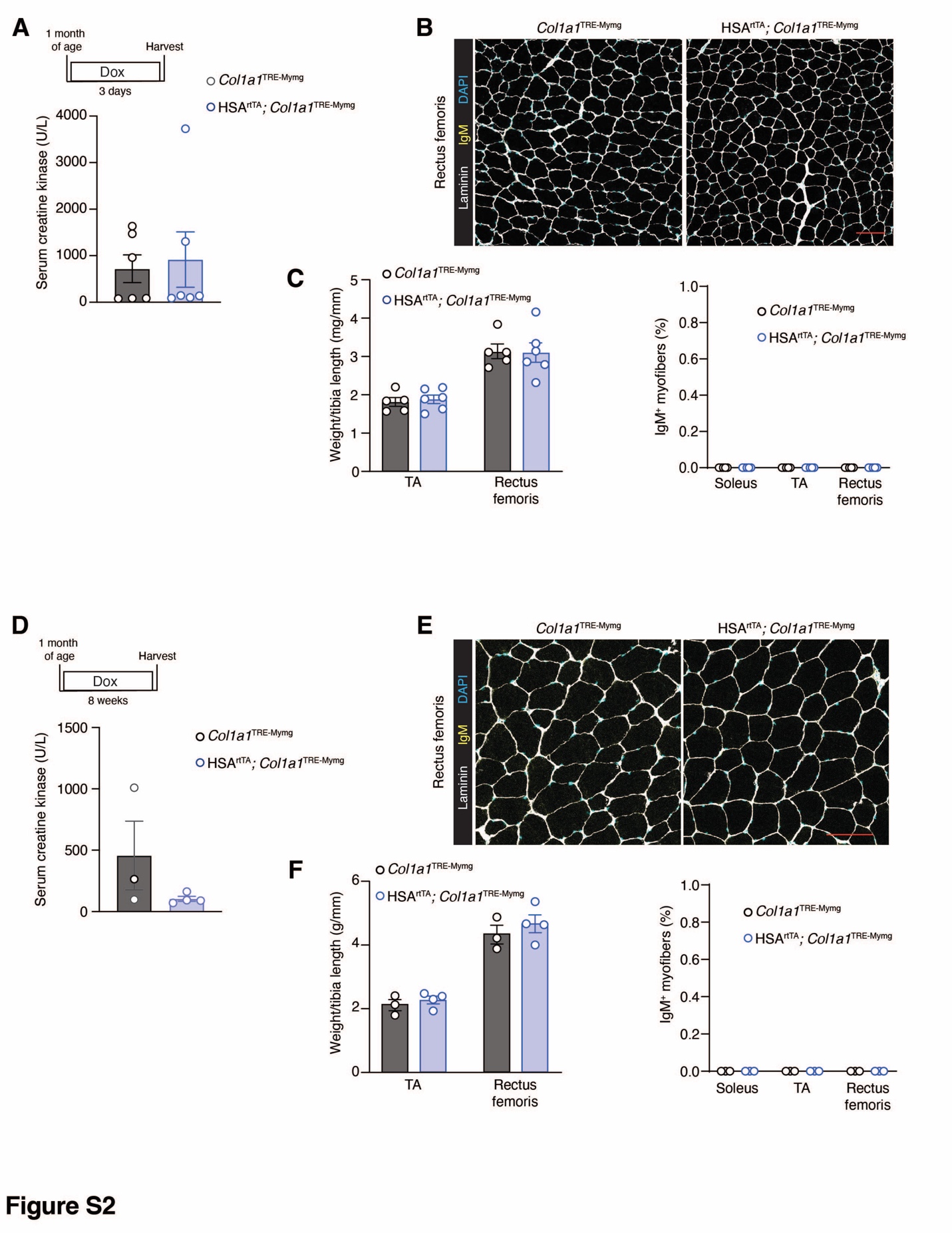
**

**Figure S2.** Myomerger expression in myofibers of iMymg mice does not lead to pathology. **A** Serum CK levels are not elevated after three days of Myomerger expression in myofibers. **B** Immunofluorescence staining for IgM after three days of Myomerger induction. Quantification of the percentage of IgM^+^ myofibers in the soleus, TA, and rectus femoris is shown below the images. Scale bar = 100 μm. **C** Muscle mass to tibia length ratios of the TA and rectus femoris are not altered after three days of Myomerger expression in myofibers. **D** Serum CK levels are not elevated after eight weeks of Myomerger expression in myofibers. **E** Immunofluorescence staining for IgM after eight weeks of Myomerger induction. Quantification of the percentage of IgM^+^ myofibers in the soleus, TA, and rectus femoris is shown below the images. Scale bar = 100 μm. **F** Muscle mass to tibia length ratios of the TA and rectus femoris muscles are not altered after eight weeks of Myomerger expression in myofibers. Statistical analyses and presentation: Data are presented as mean ± SEM; **A,C,D,F** two-tailed Student’s *t* test (within the same muscle for **C** and **F**).

**
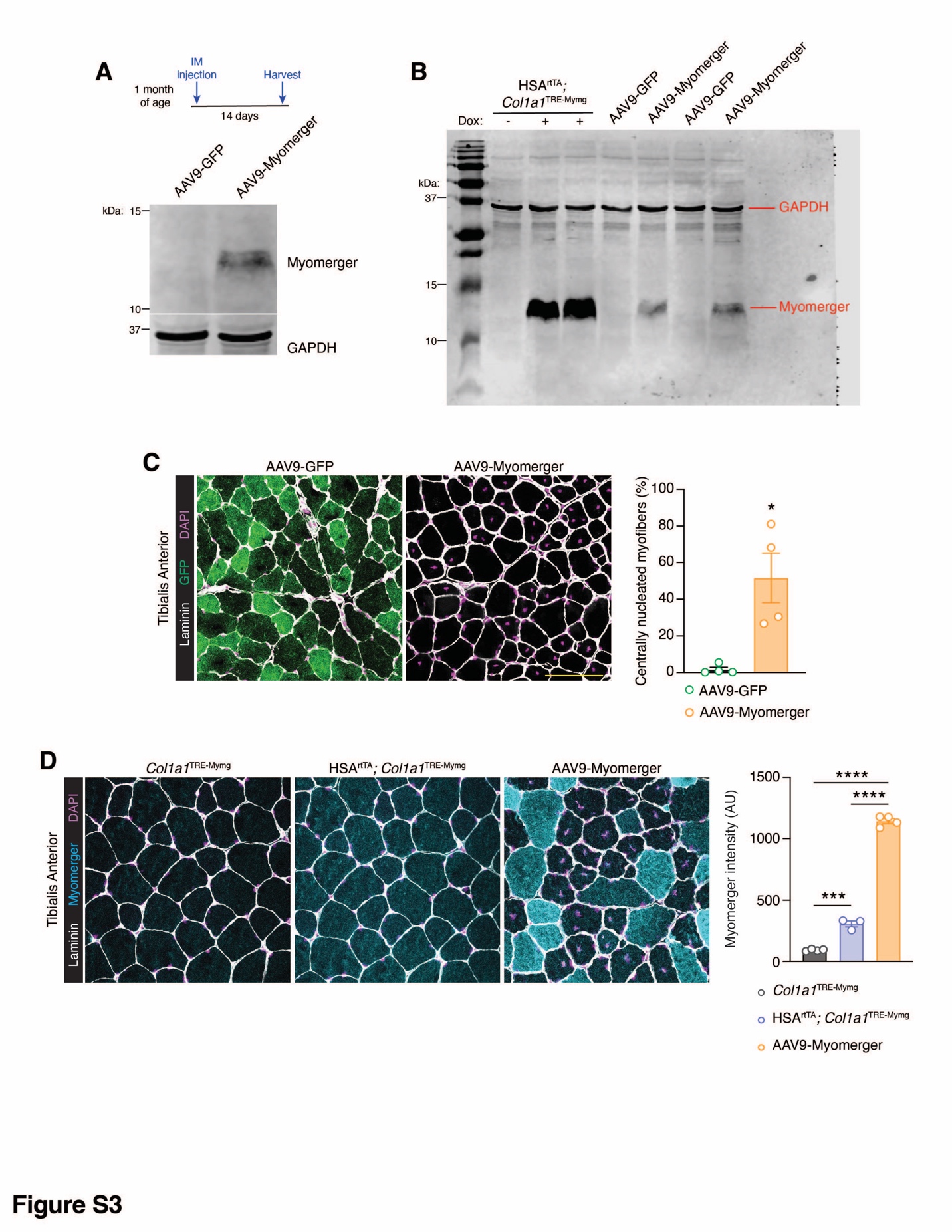
**

**Figure S3.** Elevated Myomerger expression leads to muscle regeneration. **A** Western blot for Myomerger validated transduction by AAV9-Myomerger two weeks after IM injection in the TA. **B** Western blot of Myomerger revealed higher levels of Myomerger in iMymg muscle after two weeks of dox treatment compared to AAV9-Myomerger injected muscle two weeks after IM injection. **C** Histological analysis revealed elevated levels of centrally nucleated myofibers in the TA two weeks after IM injection with AAV9-Myomerger. Scale bar = 100 μm. **D** Myomerger immunofluorescence staining of the TA revealed elevated levels of Myomerger after AAV9-Myomerger (two weeks post-injection) compared to the myofiber inducible mouse model after two weeks of dox treatment. Fluorescence intensity of Myomerger antibody staining is quantified in arbitrary units (AU). Scale bar = 100 μm. Statistical analyses and presentation: Data are presented as mean ± SEM; **C** two-tailed Student’s *t* test; **P* < 0.05; **D** one-way ANOVA with a Tukey’s post hoc test; ****P* < 0.001, *****P* < 0.0001.

**
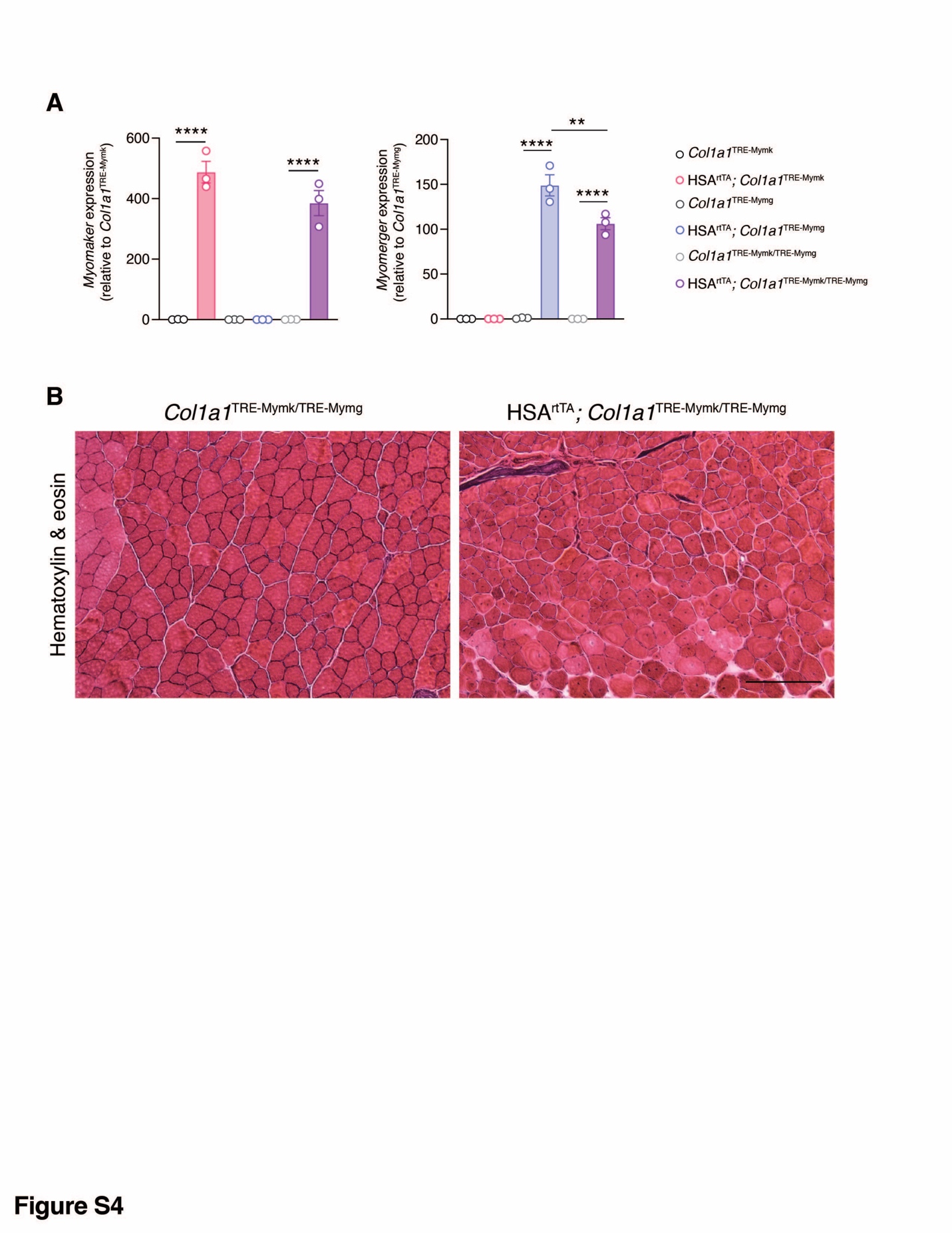
**

**Figure S4.** Myomaker and Myomerger expression in myofibers leads to muscle pathology. **A** qPCR analysis comparing *Myomaker* and *Myomerger* mRNA levels in the gastroc muscle between iMymk, iMymg, and iMymk/Mymg mice after three days of induction. **B** Representative H&E sections from the TA of *Col1a1*^TRE-Mymk/TRE-Mymg^ and HSA^rtTA^; *Col1a1*^TRE-Mymk/TRE-Mymg^ mice. Scale bar = 200 μm. Statistical analyses and presentation: Data are presented as mean ± SEM; **A** one-way ANOVA with a Tukey’s post hoc test; ***P* < 0.01, *****P* < 0.0001.
